# Quality of life as indicator of poor outcome in hemodialysis: relation with mortality in different age groups

**DOI:** 10.1186/s12882-017-0621-7

**Published:** 2017-07-06

**Authors:** I. N. van Loon, M. L. Bots, F. T. J. Boereboom, M. P. C. Grooteman, P. J. Blankestijn, M. A. van den Dorpel, M. J. Nubé, P. M. Ter Wee, M. C. Verhaar, M. E. Hamaker

**Affiliations:** 1 Dianet Dialysis Center, Brennerbaan 130, 3524 BN Utrecht, The Netherlands; 2 Department of Internal Medicine, Diakonessenhuis Utrecht, Utrecht, The Netherlands; 3 Department of Nephrology and Hypertension, University Medical Center Utrecht, Utrecht, The Netherlands; 4 Julius Center for Health Sciences and Primary Care, University Medical Center Utrecht, Utrecht, The Netherlands; 5 Department of Nephrology, VU University Medical Center, Amsterdam, The Netherlands; 6 Department of Internal Medicine, Maasstad Hospital, Rotterdam, The Netherlands; 7 Department of Geriatrics, Diakonessenhuis Utrecht, Utrecht, The Netherlands

**Keywords:** End-stage renal disease, Dialysis, Quality of life, Frailty, Geriatric nephrology

## Abstract

**Background:**

Physical, cognitive and psychosocial functioning are frequently impaired in dialysis patients and impairment in these domains relates to poor outcome. The aim of this analysis was to compare the prevalence of impairment as measured by the Kidney Disease Quality of Life- Short Form (KDQOL-SF) subscales between the different age categories and to assess whether the association of these subscales with mortality differs between younger and older dialysis patients.

**Methods:**

This study included data from 714 prevalent hemodialysis patients, from 26 centres, who were enrolled in the CONvective TRAnsport STudy (CONTRAST NCT00205556, 09–12-2005). Baseline HRQOL domains were evaluated for patients <65 years, 65–74 years and over 75 years. Multivariable Cox proportional hazards analyses were performed to assess the relation between the separate domains and 2-year mortality.

**Results:**

Emotional health was higher in patients over the age of 75 compared to younger patients (mean level 71, 73 and 77 for increasing age categories respectively, *p* = 0.02), whilst physical functioning was significantly lower in older patients (mean level 60, 48 and 40, *p* < 0.01). A low level of physical functioning (Hazard Ratio (HR) 1.72 [95%Confidence Interval (CI) 1.02–2.73]), emotional health (HR 1.85 [95% 1.30–2.63]), and social functioning (HR 1.59 [95% CI 1.12–2.26]), was individually associated with an increased 2-year mortality within the whole population. The absence of effect modification suggests no evidence for different relations within the older age groups.

**Conclusions:**

In dialysis patients, older age is associated with lower levels of physical functioning, whilst the level of emotional health is not associated with age. KDQOL-SF domains physical functioning, emotional health and social functioning are independently associated with mortality in prevalent younger and older hemodialysis patients.

**Electronic supplementary material:**

The online version of this article (doi:10.1186/s12882-017-0621-7) contains supplementary material, which is available to authorized users.

## Background

Patients with end-stage kidney disease (ESKD) are prone to accelerated aging. [1] Common underlying pathways, such as microvascular damage and inflammation, can both cause decline of kidney function and contribute to the development of impairments such as physical dependency, cognitive impairment and depression, malnutrition and comorbidity burden. [2, 3] Accumulation and interaction of these impairments across different domains ultimately results in a frailty phenotype, which renders a patient more vulnerable to external stressors and more prone to adverse events. [4] In patients over 75 years of age with a high comorbidity burden, defined as a high comorbidity score [5] or in particular involving ischemic heart disease, [6] initiation of dialysis does not prolong life in comparison to conservative care, and the hospitalization rate may be higher in dialysis patients. [5] Therefore, in a selected population, conservative care may be a good alternative to be discussed with patients and their caregivers. An adequate estimation of prognosis of ESKD patients may support this decision-making process in frail and elderly patients and may guide choices on advanced care planning such as resuscitation and surgery. However, prediction of poor outcome remains a challenge.

In addition to classic prognostic factors, such as body mass index and laboratory values such as albumin and haemoglobin, impairments in physical, cognitive and psychosocial domains have been found to independently predict poor outcome in the chronic kidney disease (CKD) population, but there is limited evidence available. [7, 8] Other studies have demonstrated that quality of life is related to survival and poor outcome in hemodialysis patients. [9, 10] The Kidney Disease Quality of Life-Short Form (KDQOL-SF) has been widely used in CKD and contains eight generic subscales that assess various aspects of health-related quality of life (HRQOL). [11] These subscales have been shown to be useful substitutes of previously validated instruments designed to assess the presence of geriatric impairments in the general population. [12, 13] Although these subscales are self-reported, previous studies have demonstrated that the subscales for physical functioning and emotional health both related well to objective and more elaborate measurements for geriatric impairment, for instance the Geriatric Depression Scale, or to muscle mass measurements. [14, 15] Therefore, these subscales may provide additional information on the prognosis of older dialysis patients. In this analysis using data from CONTRAST, in which patients were randomized to either low-flux hemodialysis or to online hemodiafiltration [16], we compared the prevalence of impairment as measured with the KDQOL-SF subscales between younger and older patients and assessed whether the relation with two-year mortality differs between younger and older patients.

## Methods

### Patients and study design

The Convective Transport Study (CONTRAST NCT00205556) is a multicentre randomized controlled trial, including patients from dialysis centres in The Netherlands (26 centres), Canada (2 centres) and Norway (1 centre). CONTRAST was conducted between 2004 and 2010 and compared the effects of low-flux hemodialysis with online hemodiafiltration (HDF) on all-cause mortality and cardiovascular events as described elsewhere. [17] Patients were eligible if undergoing hemodialysis 2 or 3 times a week, for at least 2 months, with a minimum dialysis urea Kt/V ≥ 1.2 (marker for dialysis adequacy) and were able to fully understand the study procedures. Exclusion criteria were age < 18 years, treatment by hemodiafiltration or high-flux hemodialysis within 6 months preceding randomization, severe incompliance defined as non-adherence to the dialysis prescription, a life expectancy <3 months due to causes other than kidney disease and participation in another clinical trial. The study was conducted in accordance with the Declaration of Helsinki and has been approved by the medical ethics review boards of all participating hospitals. Written informed consent was obtained from all patients prior to enrollment. [18] For the present analyses, both study arms were used collectively. The overall results from CONTRAST showed no benefit of HDF above HD on mortality risk. [16]

### Data collection

Baseline demographical data were collected including age, gender, race and educational levels. Comorbidity data included diabetes and previous cardiovascular disease (yes/no). Other clinical characteristics included cause of kidney failure, vascular access, hemodialysis dose (single pool Kt/V urea), time on dialysis, residual renal function (present yes/no), treatment time and frequency, blood pressure, body mass index (BMI) and smoking habits. The primary endpoint in the present analyses is all-cause mortality. Deaths were reported within 24 h to the data management centre by fax or email. It is important to note that after participants discontinued with the randomized treatment, they continued to be followed to assess vital status, irrespective of the reason for discontinuation. [16] Data were collected by the CONTRAST study group (which involved MLB, MPG, PJB, MAD, MJN, PTW).

### Kidney disease quality of life- short form (KDQOL-SF)

**Fig. 1:**
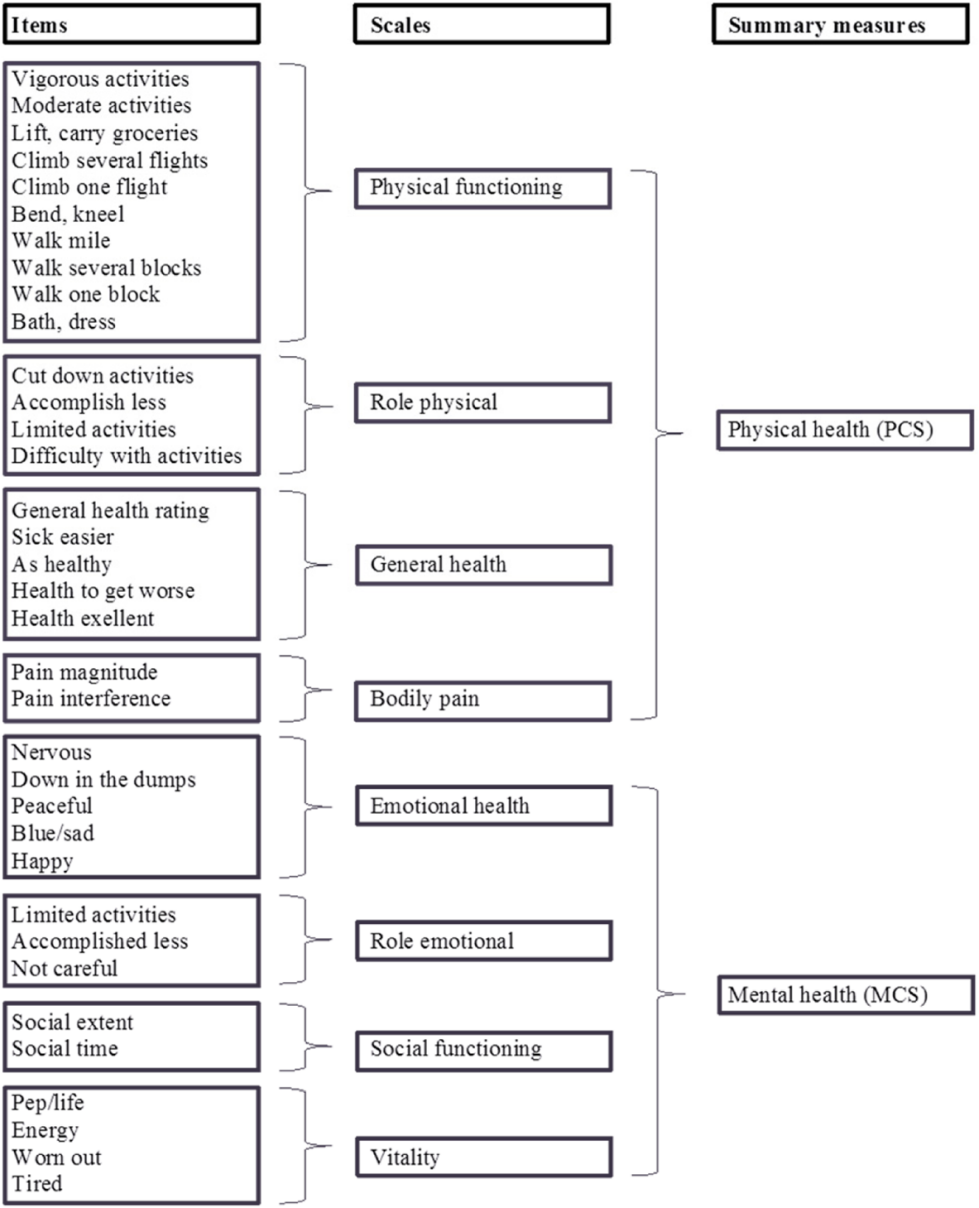
36-Item Short Form Survey Instrument (SF-36)

Quality of life was assessed at baseline with the KDQOL-SF version 1.3 (https://www.rand.org/content/dam/rand/pubs/papers/2006/P7994.pdf) as detailed previously. [18] For the present analyses, we focused on the eight generic health related quality of life (HRQOL) subscales: physical functioning, role physical, general health, bodily pain, emotional health, role emotional, social functioning and vitality (Fig. 1). All subscales have a score of 0–100, with higher scores indicating better health status. The internal consistency reliability of the generic domains of the KDQOL is moderate to high, with a Cronbach’s alpha ranging from 0.71–0.92. [11, 19, 20] These domains proved to be valid in dialysis patients by relating positively and significantly to the EuroQol (a standardized generic measure for the description of health status) [11], to the numbers of good or bad days or the self-rating of one’s life compared to people without kidney disease. [19]

### Data analysis

Data were summarized using means with standard deviation (SD), medians with interquartile ranges, or proportions when appropriate. For age we a priori defined three groups (< 65, 65–74 and ≥75 years old). All eight HRQOL domains were categorized into three groups: a score of 0–33 was defined as “low”, 34–66 as “intermediate” and 67–100 as “high”. Patients with available data on one ore more KDQOL domains were included in the analyses. Differences between the baseline characteristics of the strata were analyzed using chi-squared statistics for categorical variables and ANOVA for continuous variables. With regard to survival, chi-squared statistics were applied to compare the relation with 2-year mortality between the different categories (low, intermediate and high) per HRQOL domain and between the different age categories. Cox proportional hazards models were used to calculate hazard ratios of two-year mortality and their 95% confidence intervals (95% CI). The proportional hazards assumption was tested for each variable using a log-minus-log plot. Potential confounders were selected based on known predictors of mortality in dialysis. These factors included demographics (sex, age), treatment related items (HD vs HDF [21], time on dialysis [22]), comorbidities (diabetes mellitus [23] and cardiovascular diseases [24]), and albumin. [25] A two-tailed *p* < 0.05 was considered statistically significant. [26] A post hoc multivariate analysis was performed for the three age groups. In order to test for heterogeneity between young and old patients, we assessed whether effect modification was significantly present. Data were anonymized before analysis by the CONTRAST study group.

## Results

### Patient characteristics

The CONTRAST study included 714 patients of which 62% were male. Mean age was 64 (SD ± 14) years and 24% of the patients were over 75 years old. Median time on dialysis before entering the study, was 2 years (interquartile range 1–4 years). Table 1 summarizes the characteristics of the included patients by the three age cohorts. Information on covariates was complete for all patients, except for some missing values on diabetes (3.5% missing) and albumin (1.2% missing).

**Table 1:** Baseline characteristics

Demographics	< 65 years	65–74 years	≥ 75 years	
	(*n* = 328)	(*n* = 212)	(*n* = 174)	*p* value
Age (years)	51.9 ± 9.8	70.3 ± 2.8	79.4 ± 3.3	
Sex (% male)	60%	63%	66%	0.41
High educational status^a^	22%	17%	22%	0.30
Vascular access				
arteriovenous fistula	84%	78%	72%	0.01
graft	11%	12%	20%	0.03
central venous catheter	5%	9%	8%	0.16
Diastolic blood pressure (mmHg)^b^	80 ± 11	73 ± 12	70 ± 11	<0.001
Systolic blood pressure (mmHg)^b^	148 ± 21	148 ± 20	147 ± 24	<0.001
Time on RRT (years)	3.1 ± 3.2	2.8 ± 2.6	2.7 ± 2.3	0.16
Dialysis frequency 3×/week	95%	93%	91%	0.17
Session time (hours)	3.8 ± 0.4	3.8 ± 0.3	3.7 ± 0.4	0.02
Residual renal function^c^	48%	54%	60%	0.04
Body mass index (kg/m2)	25.2 ± 5.1	25.8 ± 4.59	25.3 ± 4.4	0.30
Current smoker	26%	16%	10%	<0.001
Diabetes Mellitus	24%	29%	21%	0.24
Cardiovascular disease	31%	52%	59%	<0.001
Kt/v urea	1.40 ± 0.22	1.40 ± 0.21	1.38 ± 0.21	0.62
Hemoglobin (mmol/L1)	7.3 ± 0.8	7.3 ± 0.8	7.4 ± 0.7	0.46
Albumin (g/L)	41.0 ± 3.9	40.2 ± 3.8	39.4 ± 3.5	<0.001
Phosphate (mmol/L)	1.74 ± 0.52	1.59 ± 0.47	1.51 ± 0.43	<0.001
Health related QoL domains				
Physical functioning	60 ± 27	48 ± 28	40 ± 28	< 0.001
Role physical	47 ± 43	41 ± 44	39 ± 44	0.10
General health	43 ± 21	44 ± 21	44 ± 19	0.26
Bodily pain	73 ± 26	69 ± 28	70 ± 28	0.44
Emotional health	71 ± 20	73 ± 21	77 ± 18	0.02
Role emotional	68 ± 43	65 ± 44	68 ± 43	0.63
Social functioning	68 ± 27	69 ± 29	67 ± 28	0.78
Vitality	54 ± 21	53 ± 23	53 ± 21	0.99

Legend: values are means +/− SD, median (interquartile range) or number (percentage), RRT renal replacement therapy, Kt/V urea fractional clearance of body water of urea, QoL quality of life

^a^University ^b^Before dialysis ^c^Residual kidney function if diuresis ≥100 ml/24 h

*P* value reflects the result of a univariable model (ANOVA or chi-square test)

### Health-related quality of life at baseline

The response rate for HRQOL was 96% (*n* = 686) for at least one domain and 87% (*n* = 620) for all HRQOL domains at baseline. Mean level of physical functioning of the oldest patients (≥ 75 years) was 20 points lower (mean ± SD: 40 ± 28) compared to the youngest group (mean ± SD: 60 ± 27) (*p* < 0.01). The distribution of the response, reflected by the standard deviation around the mean, was similar across all age groups for all domains. A decreased level of physical functioning (defined as a score < 66) was reported by 58% of the patients under 65 years old, 72% of the patients aged 65–74 years and 78% of the patients ≥75 years (*p* < 0.01). In contrast, higher levels of emotional health were associated with older age. Of the oldest patients 79% reported a good level of emotional health (defined as a score of 67–100), whilst those patients aged below 65 and those aged 65–74 years old, this was 65% and 66% respectively. The levels of the other baseline HRQOL domains (role physical, general health, bodily pain, role emotional, social functioning, vitality) were comparable between the age groups.

### Relation between quality of life domains and mortality

The incidence of all-cause mortality of CONTRAST was 12 per 100 patient years. Two-year mortality was 24%; 10% in patients under 65 years, 19% in patients between 65 and 74 years, and 40% in the patients over 75 years (*p* < 0.001). In the unadjusted analysis all domains, with the exception of the subscale role emotional, were significantly related to 2-year mortality (Table 2). The association with mortality was most distinct within the domain of physical functioning, as patients with a poor baseline level of physical functioning had a 3.6 times higher risk of dying after two years compared to those with good baseline functioning.

**Table 2:** 2-year mortality

Baseline performance		2-year mortality							
HRQoL subscales	Score	All age	*p*	< 65 year	*p*	65–74 year	*p*	≥ 75 year	*p*
Physical functioning	Poor	36%	<0.01	23%	<0.00	35%	<0.00	46%	0.12
	Intermediate	15%		6%		18%		34%	
	Good	10%		8%		5%		28%	
Role physical	Poor	24%	0.01	13%	0.16	23%	0.28	44%	0.28
	Intermediate	13%		3%		13%		46%	
	Good	15%		8%		15%		31%	
General health	Poor	26%	0.01	15%	0.17	31%	0.01	50%	0.11
	Intermediate	17%		8%		13%		37%	
	Good	14%		8%		19%		22%	
Bodily pain	Poor	32%	0.01	17%	0.24	34%	0.06	50%	0.49
	Intermediate	20%		11%		18%		37%	
	Good	17%		8%		15%		37%	
Emotional health	Poor	28%	0.04	19%	0.43	33%	0.05	50%	0.01
	Intermediate	25%		11%		28%		63%	
	Good	17%		9%		15%		33%	
Role emotional	Poor	23%	0.28	10%	0.86	27%	0.27	44%	0.51
	Intermediate	18%		12%		14%		50%	
	Good	18%		9%		17%		35%	
Social functioning	Poor	30%	0.02	20%	0.12	30%	0.36	50%	0.02
	Intermediate	22%		8%		19%		50%	
	Good	17%		10%		18%		29%	
Vitality	Poor	30%	0.02	15%	0.44	24%	0.69	58%	0.05
	Intermediate	18%		9%		19%		36%	
	Good	17%		9%		17%		31%	

Score: Poor (0–33), Intermediate (34–66), Good (67–100). *P* value reflects the result of a univariable model (ANOVA)

When focusing on the different age categories, the association with mortality and impaired HRQOL domains was also noted. In the unadjusted analyses, physical functioning was significantly associated with 2-year mortality risk in patients <65 and 65–74 years old. For patients over the age of 75, a lower level of physical functioning was also associated with a higher mortality rate, although not statistically significant. In the age group 65–74 years old, good levels of general health and emotional heath were associated with better 2-year survival, whilst a good level of emotional health, social functioning and vitality were associated with better 2-year survival in the oldest population.

After adjustment for age and other relevant patient and treatment factors, a decreased level (defined as a score of ≤66) of physical functioning (hazard ratio (HR) 1.72 [95% confidence interval (CI) 1.02–2.73]), emotional health (HR 1.85 [95% CI 1.30–2.63]) and social functioning (HR 1.59 [95% CI 1.12–2.26]) were significantly associated with mortality during a follow-up of two years (Table 3). All other domains were not significantly related to 2-year mortality. In addition, physical functioning and emotional health were associated with 2-year mortality in the 65–74 year group and general health, whilst emotional health and social functioning were associated with 2-year mortality in the oldest group. The magnitude of the relation between HRQOL domains and mortality does not appear to differ with aging as indicated by the absence of significant effect modification (data not shown). The HRs for the association between the different domains and mortality for the whole group and the subanalyses may be found in Table 3.

**Table 3:** Cox adjusted 2 year-mortality for decreased HRQOL domains^a^

Domain	HR^b^	95% CI	*p*
Physical functioning	1.72	(1.02–2.73)	0.02
Role physical	1.37	(0.92–2.03)	0.12
General health	1.50	(0.84–2.68)	0.17
Bodily pain	1.37	(0.97–1.95)	0.07
Emotional health	1.85	(1.30–2.63)	0.001
Role emotional	1.27	(0.87–1.84)	0.22
Social functioning	1.59	(1.12–2.26)	0.01
Vitality	1.37	(0.91–2.06)	0.13
a. Age < 65 year			
Physical functioning	0.76	(0.33–1.74)	0.51
Role physical	1.07	(0.49–2.31)	0.87
General health	1.01	(0.33–3.65)	0.89
Bodily pain	1.07	(0.50–2.27)	0.86
Emotional health	1.02	(0.49–2.16)	0.95
Role emotional	0.76	(0.33–1.47)	0.51
Social functioning	0.97	(0.47–2.00)	0.92
Vitality	0.84	(0.35–2.01)	0.70
b. Age 65–74 years			
Physical functioning	5.70	(1.74–18.69)	0.04
Role physical	1.49	(0.71–3.12)	0.29
General health	0.99	(0.41–2.38)	1.00
Bodily pain	1.63	(0.84–3.13)	0.15
Emotional health	2.41	(1.26–4.60)	0.01
Role emotional	1.49	(0.76–2.91)	0.24
Social functioning	1.41	(0.74–2.70)	0.30
Vitality	1.23	(0.60–2.55)	0.57
c. Age ≥ 75 years			
Physical functioning	1.47	(0.73–2.97)	0.28
Role physical	1.45	(0.81–2.63)	0.21
General health	2.59	(0.92–7.32)	0.07
Bodily pain	1.30	(0.77–2.18)	0.33
Emotional health	2.11	(1.22–3.64)	0.01
Role emotional	1.43	(0.80–2.54)	0.22
Social functioning	2.01	(1.20–3.52)	0.01
Vitality	1.67	(0.89–3.14)	0.11

Adjusted for age, sex, treatment modality, time on dialysis, cardiovascular disease, diabetes mellitus, albumin

^a^Decreased score defined as 0–66. ^b^Reference group: patients with a score of 67–100

When we applied a multivariate analysis using a cut-off value of 50, which approaches the value of the domains with the lowest means, we found the same results for the three domains. (Additional file 1). A higher chosen cut-off value of 75 only resulted in a significant relation with 2-year mortality for the domain emotional health (HR 2.15 [95% CI 1.12–4.14] *p* = 0.02). When we insert the domains as continuous variables, we found all HRQOL domains were significantly associated with 2-year mortality (Additional file 1).

## Discussion

Due to the aging of society, more and more patients will develop ESKD and consequently the dialysis population will be increasingly characterized by patients with impairments such as functional dependencies, cognitive decline and psychosocial impairment. [4] In our study, a significant lower level of physical functioning was seen in the advanced age groups, and the lowest level of phyical functioning was seen in patients aged over 75 years old. Although the HRQOL emotional health subscale, including five questions relating to mood, was affected in all age groups, it was relatively preserved in dialysis patients over 75 years old in comparison to younger dialysis patients. Levels of the other baseline HRQOL domains were comparable between the age groups. Finally, this analysis of CONTRAST shows that an impaired level of physical functioning, emotional health and social functioning domains are strongly and independently associated with 2-year mortality independent of age and other confounders. In older dialysis patients this knowledge may contribute to advanced care planning.

The results of this study add to a growing body of evidence which shows that impairments across the physical and psychosocial domains are related to poor outcome in the CKD and dialysis population. [7, 27] In the Dialysis Outcomes and Practice Patterns Study (DOPPS), among 10.030 patients with a mean age of 60.5 ± 15.2 years) all eight subscales of the KDQOL-SF were shown to be related to mortality with an adjusted RR per 10-point lower HRQOL score varying from 1.03 (role physical) to 1.10 (physical functioning). [10] Although the individual HRQOL domains have also been found to be valid in the elderly, [12] evidence on the use of the separate domains is limited within the elderly population. In the CONTRAST population, emotional health was shown to be significantly better in patients over 75 years of age compared to younger patients. Others have shown that, for patients over 75 years old, the mental component score (MCS), including domains role emotional, social functioning and vitality, was comparable for dialysis patients and the general elderly population. [28] The relatively good emotional health in elderly patients may reflect lower expectations and a higher level of satisfaction with being alive despite functional disabilities. [29] For the physical functioning subscale, the low mean score in elderly patients is in accordance with previous studies which document a high rate of physical impairment and functional dependencies within this population. [30, 31] Prior research has shown a graded response between the Physical Component Score (PCS) and mortality for increasing age strata, but this relationship was lost in the oldest age category (85 years old or older). [32] The lack of discriminatory power in the highest age group was suggested to be due to competing mortality risk in the elderly. In the CONTRAST population, the 2-year mortality rate of the oldest age group was 46% for patients with low physical functioning compared to 35% and 28% for those with intermediate impairment and good physical functioning respectively (*p* = 0.12). This is much higher than the expected chance of mortality in the general (non ESKD) elderly (≥ 75 years) population. Based on the data of the national life tables available at Statistics Netherlands, the expected 2-year mortality for an age- and sex-matched population would be 11.6% (± 6.1). [33] Potentially, the relation between physical functioning and mortality in the oldest group was not statistically significant due to small sample size. Similarly, the small sample size for subgroup analyses most likely accounts for the lack of statistical significance between the domain social functioning and two-year mortality in the 65–74 year age group. However, the effect sizes of the multivariate analyses of the domains physical functioning and social functioning for the older age groups were in the same direction as they were in the primary mortality analysis. In addition, the interaction term of age was not statistically significant, and thus the relation of the HRQOL domains and mortality does not appear to be different across age groups. [34]. Appreciating the limitation of the sample size, we can conclude that the domains physical functioning, emotional health and social functioning are also related to two-year mortality in patients aged over 65 years.

The KDQOL domains *emotional health* and *physical functioning* have been used separately as substitute tests of impairment in physical performance and mood. The subscale emotional health has been used within the dialysis population to detect depressive symptoms and was shown to be related to the Beck Depression Inventory, a self-reported inventory which has been extensively used in order to detect depression. [15] The physical functioning subscale showed to be significantly related with more elaborate measurements, such as gait speed, and was considered a valid substitute test of physical performance. [14] Given the association between these quality of life domains and mortality, assessment of these domains may aid in establishing patients’ biological reserves and identifying vulnerable ESKD patients. It might help in selecting patients who would benefit from (preventive) interventions, such as physical exercise [31] and (pharmacological) treatment of depression. [35] In addition, this information might contribute in providing a long-term care plan, counseling and in end-of-life discussions, especially in the elderly (patients over 75 years old), for whom such decisions are frequently challenging. However, future research should assess whether these less elaborate tests could be beneficial here.

In addition to the previous mentioned limitation, this study has raised some other issues, which need to be addressed. The prevalence of impairment is likely an underestimation of what is found in the general dialysis population. Patients in the poorest health and with the highest risk of death may be either excluded from participation in the trial or decided not to participate. In addition, CONTRAST was conducted in three countries with a high quality health care system and a good financial and social network for the elderly. If we had corrected for multiple testing using a more stringent *p*-value (e.g. Bonferroni method 0.05/24 = 0.002), only physical functioning (univariate analysis) and emotional health (multivariate analysis) would have been statistically significant. However, our analyses were of explorative nature instead of clinical trial analyses, and a too stringent significance level may cause loss of relevant information regarding potential relations. The consistently statistically significant relations of all but one domain with mortality, made us decide not to discard these relations, as there are more significant relations than would have been expected based on change only. Therefore we decided to base our conclusions on significant relations on *p*-values <0.05 thereby not allowing for correction for multiple testing. We have chosen the covariates based on clinical relevance, however, we can not rule out residual confounding, such as the influence of socioeconomic background. Finally, we arbitrarily divided the HRQOL domains into tertiles, and considered only the highest tertile to be “good”, thus enhancing interpretation by the clinician. Based on the means and SD in our cohort (Table 1), we can conclude that for roles of physical, general health and vitality the cut-off value of 66 for a normal score might be too high. Consequently this may have resulted in underestimation of the effect on mortality. However, although the height of the cut-off value is under debate, our results show the robustness of the association with 2-year mortality for the domains physical functioning, emotional health and social functioning.

## Conclusion

In dialysis patients, advanced age is associated with lower levels of physical functioning, while levels of emotional heath are not associated with age. As measured with the KDQOL-SF, the subscales physical functioning, emotional health and social functioning are strongly associated with 2-year mortality independent of age. Given this association, assessment of physical, mental and social domains may be of help in identifying frail patients at risk for poor outcomes and could form a starting point for preventive interventions. The subscales could also be useful in treatment decisions and end-of-life discussions, particularly in the elderly (patients over 75 years old) where such decisions are frequently challenging. This should be explored in more detail through future research.

## Additional file

Additional file 1: Table S1. Cox adjusted 2 year-mortality for decreased HRQOL domains. Lower cut-off value 50. Decreased score defined as 0–49. Reference group: patients with a score of 50–100. Table S2. Cox adjusted 2 year-mortality for HRQOL domains. Domains included as continuous variables (DOC 39 kb)

## Abbreviations

ANOVA: Analysis of variance
CI: Confidence Interval
CKD: Chronic kidney disease
CONTRAST: CONvective TRAnsport STudy
ESKD: End-stage kidney disease
HD: Hemodialysis
HDF: Hemodiafiltration
HR: Hazard Ratio
HRQOL: Health-related quality of life
KDQOL-SF: Kidney Disease Quality of Life- Short Form
MCS: Mental component score
PCS: Physical component score
SD: Standard deviation
