# Supplementary material for: Quality of life as indicator of poor outcome in hemodialysis: relation with mortality in different age groups
Source: BMC Nephrol. 2017 Jul 6;18:217. doi: 10.1186/s12882-017-0621-7 (PMC5498985; doi:10.1186/s12882-017-0621-7)
Supplement: Additional file 1: Table S1. — Cox adjusted 2 year-mortality for decreased HRQOL domains. Lower cut-off value 50. Decreased score defined as 0–49. Reference group: patients with a score of 50–100. Table S2. Cox adjusted 2 year-mortality for HRQOL domains. Domains included as continuous variables (DOC 39 kb) [file 12882_2017_621_MOESM1_ESM.doc]

**Appendix 1**

| **Table 3d. Cox adjusted 2 year-mortality for decreased HRQOL domains^*^** | | | |
| --- | --- | --- | --- |
| Domain | HR^#^ | 95%CI | p |
| Physical functioning | 2.13 | (1.46-3.13) | < 0.001 |
| Role physical | 1.53 | (1.05-2.23) | 0.03 |
| General health | 1.62 | (1.12-2.33) | 0.01 |
| Bodily pain | 1.51 | (1.06-2.16) | 0.02 |
| Emotional health | 1.57 | (0.97-2.53) | 0.07 |
| Role emotional | 1.31 | (0.89-1.91) | 0.17 |
| Social functioning | 1.56 | (1.06-2.30) | 0.02 |
| Vitality | 1.74 | (1.22-2.48) | 0.002 |

Adjusted for age, sex, treatment modality, time on dialysis, cardiovascular disease, diabetes mellitus,

albumin.

* Decreased score defined as 0-49. ^#^Reference group: patients with a score of 50-100

| **Table 3e. Cox adjusted 2 year-mortality for HRQOL domains^*^** | | | |
| --- | --- | --- | --- |
| Domain | HR^#^ | 95%CI | p |
| Physical functioning | 1.013 | (1.006-1.019) | < 0.001 |
| Role physical | 1.005 | (1.000-1.009) | 0.032 |
| General health | 1.014 | (1.016-1.033) | 0.001 |
| Bodily pain | 1.008 | (1.002-1.013) | 0.014 |
| Emotional health | 1.012 | (1.004-1.020) | 0.003 |
| Role emotional | 1.003 | (0.999-1.007) | 0.106 |
| Social functioning | 1.009 | (1.003-1.015) | 0.002 |
| Vitality | 1.011 | (1.003-1.019) | 0.005 |

Adjusted for age, sex, treatment modality, time on dialysis, cardiovascular disease, diabetes mellitus,

albumin.

* per point increase
